# Antimicrobial Resistance, a Global Challenge

**DOI:** 10.30699/ijp.2025.2056060.3444

**Published:** 2025-08-15

**Authors:** Alireza Abdollahi, Mohammad Reza Jalali Nadoushan

**Affiliations:** 1 *Department of Pathology, School of Medicine, Imam Khomeini Hospital Complex, Tehran, Iran*; 2 *Associate Editor, Iranian Journal of Pathology, Tehran, Iran*; 3 *Dept. of Pathology, Shahed University, Tehran, Iran*; 4 *Editor-in-Chief, Iranian Journal of Pathology, Tehran, Iran*

Antibiotic resistance has become one of the greatest healthcare and therapeutic challenges in hospitals, posing a serious threat to the health of both patients and medical staff (1). As bacteria and other organisms that are capable of resisting antibiotics multiply and spread, treating many hospital-acquired infections becomes increasingly complex and difficult. This phenomenon is exacerbated by the inappropriate and excessive use of antibiotics in hospitals, particularly in intensive care units, leading to severe complications, including mortality caused by drug-resistant infections (2). Microbial resistance can result in prolonged hospitalization time, high treatment costs, and ultimately, death among patients who contract resistant infections. Therefore, identifying and controlling this resistance, especially in hospital settings, requires immediate attention and action. The World Health Organization has recently identified three categories of pathogens, namely: critical, high, and medium priority, according to the need for new antibiotics (3).

## 1. Concept and Importance of Antibiotic Resistance

Antibiotic resistance refers to the reduced or complete loss of effectiveness of antibiotics against bacteria. Bacteria use various mechanisms to counteract these drugs when exposed to them. Every year, at least 700,000 people succumb to AMR (Antimicrobial resistance). If this uncontrolled pattern continues, a cumulative cost of US $100 trillion will develop by 2050, undermining the economy, which will be comparable to the 2008 financial crisis. Annually, 33,000 people will die in the European Union and European Economic Area (EU/EEA) due to an infection with a resistant bacterial strain by 2050. The hospital stays for patients with AMR average around 13 days, causing an additional 8 million hospital days annually, costing up to US $29,000 per patient.

Globally, about 500,000 new cases of multidrug-resistant tuberculosis (MDR-TB) are diagnosed yearly. In 2018, 87% of new TB cases occurred in 30 countries in Asia, Africa, and Latin America

Most of the ESKAPE pathogens (*Enterococcus faecium, Staphylococcus aureus, Klebsiella pneumoniae, Acinetobacter baumannii, Pseudomonas aeruginosa*, and *Enterobacter species*), the leading causes of life-threatening nosocomial infections amongst critically ill and immunocompromised individuals, are multidrug-resistant isolates, and these pose the most significant challenges in clinical practice (4).

## 2. Growing Trend of Antibiotic Resistance in Hospitals

One of the key factors contributing to the rise of antibiotic resistance in hospitals is the inappropriate and excessive use of antibiotics (5).

In hospitals, the concentration of patients with various health issues and weakened immune systems creates an ideal environment for the growth and spread of antibiotic-resistant bacteria. This leads to a higher prevalence of drug-resistant infections, making treatment more challenging.

Recent studies show that the increased incidence of antimicrobial resistance could be attributed to the excess use of antimicrobial agents during the coronavirus disease 2019 (COVID-19) pandemic¸ which is related to the increase in resistance trend for 2021 and 2022 in E. coli, P. aeruginosa, S. aureus, and E. faecium. Patients with COVID-19 have been vulnerable to other secondary infections owing to multiple comorbidities with severe COVID-19, prolonged hospitalization, and severe acute respiratory syndrome coronavirus 2 (SARS-CoV-2)-associated immune dysfunction. These hospital patients have often acquired secondary bacterial infections, which can increase the odds of developing AMR (3).

## 3. Consequences of Increasing Antibiotic Resistance

Antibiotic resistance can significantly impact the treatment process for patients. Some of the key consequences include:

- **Increased Mortality**: When infections resistant to antibiotics cannot be treated, the risk of death increases, especially in cases where the immune system is already weakened.
- **Higher Treatment Costs**: Treating antibiotic-resistant infections often requires more expensive drugs, and the duration of hospital stays may also be prolonged.
- **Spread of Diseases**: Antibiotic resistance can lead to outbreaks of untreatable infections in healthcare settings, resulting in further spread of diseases and an increased need for intensive care and hospitalization.

## 4. Concept of Antibiotic Stewardship

Antibiotic stewardship refers to a set of strategies, policies, and actions aimed at reducing unnecessary and excessive antibiotic use, thereby preventing antibiotic resistance. The main goal is to control usage carefully, prevent the selection of resistant bacteria, and ultimately improve patient outcomes. There are studies demonstrating that long-course antibiotic regimens are non-superior to short-course antibiotic regimens.

However, in a survey of 113 French ICUs, only 54% of respondents stated that they followed local antibiotic protocols, and 43% were familiar with the term antimicrobial stewardship (6). Therefore, it is critical to focus on the means to implement ASP.

## 5.1. Importance of Antibiotic Stewardship in Hospitals

### 5.1.1. Preventing Antibiotic Resistance

One of the most important reasons for implementing antibiotic stewardship in hospitals is to prevent antibiotic resistance By closely monitoring and limiting antibiotic use, the development and spread of resistance can be prevented (7).

## 5.1.2. Reducing Side Effects

Overuse of antibiotics can lead to significant side effects, including gastrointestinal issues, fungal infections, kidney or liver damage, and drug allergies. 

## 5.1.3. Lowering Healthcare Costs

Excessive use of antibiotics is not only harmful to patients but also significantly increases healthcare costs. Treating antibiotic-resistant infections often requires more expensive drugs, and the length of hospital stays tends to be longer. Therefore, implementing antibiotic stewardship strategies can reduce overall hospital costs (7).

## 5.1.4. Improving the Quality of Healthcare

Proper and efficient use of antibiotics improves the quality of healthcare in hospitals. When infections are effectively treated and the risk of resistance is minimized, patients recover faster, and mortality rates from infections decrease. This is particularly important in intensive care units, where patients often have weaker immune systems.

## 6. Steps for Implementing Antibiotic Stewardship in Hospitals

To achieve the goals of antibiotic stewardship, hospitals must undertake a series of multidimensional actions. In this regard, the following steps are particularly important:

## 6.1. Assessment of Antibiotic Use

The first step in implementing an antibiotic stewardship program is to carefully evaluate and review antibiotic use within the hospital. This assessment includes examining the number of antibiotic prescriptions, the types of drugs prescribed, and the duration of treatment. Such evaluations are essential to identify cases of unnecessary or excessive antibiotic use.

## 6.2. Training Healthcare Staff

Educating healthcare staff (doctors, nurses, and pharmacists) about the risks of antibiotic resistance and the correct principles of prescribing antibiotics is another critical component of these programs. Healthcare workers should have a clear understanding of when to prescribe antibiotics, how to select the appropriate drugs, and how to use them optimally.

## 6.3. Microbiological Analysis

The use of precise microbiological tests and bacterial cultures to identify the type of bacteria causing infections and their susceptibility to antibiotics is highly important. This information can assist in selecting the most effective antibiotics and reducing the likelihood of unnecessary drug use.

## 6.4. Monitoring Programs and Feedback

Establishing monitoring systems to track antibiotic prescriptions and provide feedback to doctors and other members of the medical team is considered one of the most effective methods for managing antibiotic use. These monitoring efforts, especially in cases where antibiotics are prescribed without thorough evaluation, can prevent errors and ensure that only necessary treatments are administered.

By implementing these strategies, hospitals can play a vital role in mitigating the growing threat of antibiotic resistance and ensuring better health outcomes for patients.

In the end, it can be stated that Antibiotic resistance has become a global challenge, particularly in hospitals, requiring special attention and precise measures. Adhering to proper guidelines for prescribing antibiotics and implementing antibiotic stewardship programs in hospitals can effectively prevent the rise of antibiotic resistance and reduce mortality caused by drug-resistant infections. In this regard, education, monitoring, and strict control over antibiotic prescriptions should be prioritized as key components in any healthcare setting.
